# Commentary: locating the restriction point

**DOI:** 10.1186/s13008-023-00085-8

**Published:** 2023-02-10

**Authors:** Robert F. Brooks

**Affiliations:** Molecular and Clinical Sciences Research Institute, St George’s University of London, Mailpoint J2A, Cranmer Terrace, SW17 0RE London, UK

**Keywords:** Restriction point, Quiescence, Mitogen stimulation, Retinoblastoma protein (RB), Cyclin-dependent kinases 4 and 6 (CDK4,6), Cyclin-dependent kinase 2 (CDK2), Cyclin D, Cyclin E, Palbociclib, Cell cycle commitment

## Abstract

Attempts to map the Restriction Point in the mammalian cell cycle typically involve stimulating quiescent cells with mitogens for increasing intervals, removing the stimulus and then determining the proportion of cells that reach S phase at some point later. This “fixed point” estimate assumes that further cell cycle commitment ceases as soon as the stimulus is removed. In fact, kinetic analysis shows that the probability of cell cycle commitment does not fall back to its initial low value, immediately after a pulse of mitogens, but may instead remain slightly elevated for some while afterwards, compared to the starting quiescent population. Thus, cells entering S phase after a brief exposure to mitogens are not those that pass the Restriction Point early. Rather, they represent cells that continue on to S phase as a result of this residual, low probability of cell cycle commitment. Instead, the mitogen-regulated process(es) affecting the probability of cell cycle commitment are much closer to the start of S phase itself. Since the acquisition of (apparent) mitogen independence is such a poor indicator of the timing of cell cycle commitment, it is argued that a better measure is the point of insensitivity to CDK4,6 inhibitors such as palbociclib, which indicates when hyperphosphorylation of the Retinoblastoma Protein, RB, ceases to be dependent on mitogen-signalling pathways regulating CDK4,6/cyclin D activity.

## Background

In mammalian cells, the Restriction Point is usually taken to be an abrupt, all or none transition in G1 of the cell cycle when cells become independent of further mitogenic stimulation for entry into S phase and mitosis [1–3]. This point, analogous to START in yeasts [4], is widely considered to mark an important commitment decision, control of which is defective in most cancers [3, 5].

Rather surprisingly, estimates of when cells cross the Restriction Point and become mitogen-independent vary widely, even in the same cell type. With continuously cycling Swiss 3T3 cells (immortal mouse fibroblasts), early studies by Yen and Pardee [6] placed the Restriction Point around 3 h after mitosis (in G1), a couple of hours before the start of S phase. Later, time-lapse observations by Zetterberg & Larsson (also of cycling 3T3 cells) similarly suggested that mitogen-independence was acquired 3–4 h after mitosis, but very precisely and abruptly in *all* cells [7]. In contrast, Spencer et al. [8] found that Swiss 3T3 cells generally became mitogen-independent much more gradually, 6–10 h after mitosis. Moreover, around 20% of the population were already mitogen-independent from birth, arresting only after completing the next mitosis. In many other rapidly proliferating (immortal) lines, this was even more marked, with the majority of the cells in early G1 failing to arrest after serum withdrawal. In these cases, it would seem that commitment had already occurred before birth, in the previous cell cycle [8, 9]. This raises questions as to the universality of a Restriction Point in G1 [10].

## Restriction point passage after mitogen-stimulation of quiescent cells

For cells re-entering the cycle from quiescence, Restriction Point timing is usually assessed by restoring mitogens to starved cells for pulses of increasing duration and determining the proportion of cells that have entered S phase at a fixed point some time later, typically 20 h or more after the start of stimulation (e.g. [11–13]). When this is done, the fraction of cells in S phase rises gradually with the duration of stimulation suggesting a highly asynchronous passage of the Restriction Point. For some cells, withdrawal of mitogens after the end of the pre-replicative lag (at a time when other cells have already started DNA synthesis) prevents entry into S phase, suggesting that, for these cells, the Restriction Point is late, close to the G1/S transition. With other cells, only a short serum pulse, for as little as two hours, is required to trigger subsequent entry into S phase, suggesting early passage of the Restriction Point, many hours before the start of DNA synthesis. However, this experimental strategy contains the unacknowledged assumption that further cell cycle commitment ceases as soon as the growth stimulus is removed. This assumption is rarely validated. To do so requires a much fuller kinetic analysis than is usually done.

**Fig. 1:**
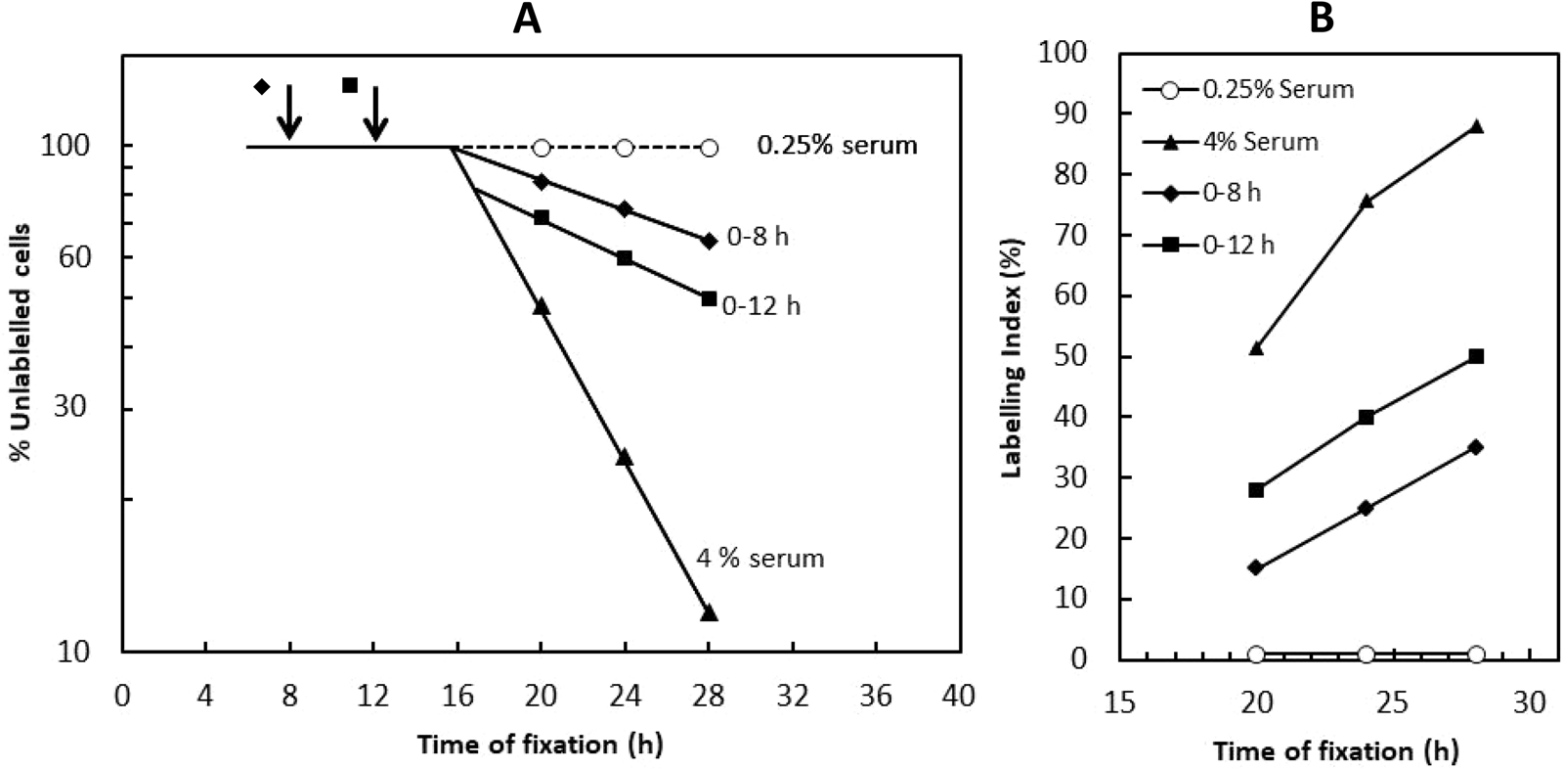
Continued passage of the Restriction Point after a serum step-down **A** Schematic adapted from Fig. 2b of [14]. Serum-starved Swiss 3T3 cells were stimulated with 4% serum from t = 0 and stepped down to 0.25% serum at either 8 h (⬥) or 12 h (◼), see arrows. Control cells remained in 4% serum throughout (▲) or were transferred to 0.25% serum from the start (○). Labelling with ^3^H-thymidine was continuous. Cells were fixed at the times indicated and the labelling index determined. The data were plotted on a logarithmic scale as the fraction of cells not yet labelled with ^3^H-thymidine, i.e. those still in G0/G1. The indicative points shown are representative for cells fixed at 20, 24 or 28 h only. For the full dataset, see [14] **B** ^3^H-thymidine labelling index for cells fixed at 20, 24 or 28 h. Alternative plot from the experiment in panel **A**, showing continued entry into S phase after the step-down

The value of such kinetics is illustrated by the experiment shown in Fig. 1 (redrawn from [14]). Here, quiescent Swiss 3T3 cells were stimulated maximally with high serum (4%, in this case), either continuously, or for intervals of 8 or 12 h, before returning to low serum (0.25%, a non-stimulatory level allowing cell survival). Labelling with ^3^H-thymidine was continuous. For the full set of data points see the original paper [14]; points for cells fixed at 20, 24 or 28 h are reproduced here for illustrative purposes (Fig. 1).

With an 8-hour serum pulse, the labelling index at 20 h was 15% (Fig. 1B). This is usually taken to indicate that 15% of the cells had passed the Restriction Point by the end of the 8-hour pulse. After the step-down, it is generally assumed that no further cells would pass the Restriction Point, in the absence of stimulation, in which case, the labelling index would be expected to remain the same for cells fixed at later time points. In fact, the labelling index rose from 15% at 20 h to 25% at 24 h and to 35% after 28 h (Fig. 1B). Similarly, with a 12-hour serum pulse, the labelling index rose from 28% at 20 h, to 40% at 24 h, and to 50% at 28 h (Fig. 1B). This increase indicates that cells were continuing to enter S phase (and therefore pass the Restriction Point), long after the serum stimulus had been withdrawn, albeit at a low rate (Fig. 1A). By the usual definition of the Restriction Point, this should not happen.

The idea that further cell cycle commitment ceases completely as soon as the growth stimulus is removed is, however, to misunderstand the processes underlying commitment decisions. Even in deeply quiescent populations, the S phase labelling index is never zero. A few cells continue to enter S phase at all times but with a very low probability. When mitogens are restored, the probability of entering S phase increases, after a lag, and cells do so asynchronously over many hours. Whether this is because commitment is due to a single probabilistic transition as suggested by Smith & Martin [15], or to multiple processes [16, 17] is immaterial. From a cellular perspective, commitment to enter into S phase is clearly stochastic [8, 18]. At the population level, this is best described empirically by a *rate* of entry into S phase from G1 rather than as the number of cells to have reached S phase at an arbitrary fixed point in time.

In these experiments, when the serum step-down was made near to or at any time after the end of the pre-replicative lag, the rate of entry into S phase declined abruptly 4–5 h later [14]. This suggests that the mitogen-regulated step controlling commitment (the Restriction Point) is located no more than 4–5 h before the G1/S transition, for these cells and conditions. However, importantly, following serum step-down, the probability of entry into S phase did not return to the very low level of unstimulated control cells. Rather, it remained elevated (despite rinsing with serum-free medium), with cells continuing to enter S phase, albeit at a low rate (Fig. 1A). This may be due in part to the difficulty of removing serum growth factors from a culture through adsorption to the extracellular matrix, or to continued signalling from internalised growth factor-receptor complexes, or to long-term physiological changes induced by the serum pulse, such as increases in RNA and protein content. Whatever the cause, when the serum step-down was made more than 4–5 h *before* the end of the pre-replicative lag (arrows in Fig. 1A), it was precisely this residual rate of entry into S phase that was responsible for the apparent commitment measured at a fixed time later. Thus, in this experiment, the cells that entered S phase after an 8 or 12 h serum pulse had not passed the Restriction Point during the pulse. Rather, they did so *after* the step-down, because the probability of commitment remained elevated compared to the initial quiescent population prior to stimulation. Of course, with other cell types or conditions, the rate of entry into S phase may fall more completely after withdrawal of mitogens, such that the number in S phase remains the same when sampled at different time points. This, however, has to be demonstrated before early passage of the Restriction Point can be taken to have occurred.

To circumvent the technical difficulties of fully reversing serum stimulation by medium change, an alternative approach is to use a MEK (MAP2K1) inhibitor to block the key RAS-MAPK pathway (e.g. [19]). However, serum growth factors stimulate other signalling pathways besides the RAS-MAPK pathway, such as the PI3K pathway, or the activation of non-receptor kinases of the Src family that (for example) down-regulate the CDK inhibitor p27 [20, 21]. These additional pathways would be unaffected by MEK inhibition and could well contribute to a significant residual level of stimulation, similar to that persisting after serum step-down, allowing slow, continued passage of the Restriction Point after RAS-MAPK pathway blockade.

As already noted, the sharp reduction in the rate of entry into S phase seen 4–5 h after a serum step-down (Fig. 1A and [14]), would seem to suggest that the growth factor-regulated step in cell cycle commitment (the Restriction Point) was 4–5 h before the start of DNA synthesis, for those cells and conditions. This, however, is a *maximum* estimate. Growth factors activate signal transduction pathways leading, among other things, to the induction of gene expression. This in turn can lead to further gene expression. Clearly, it takes a finite time for growth factor addition or removal to alter the expression of the relevant component(s) involved in cell cycle commitment. Due to this delay, the actual point of commitment will lie downstream of the step-down, closer to the start of S phase. That this is indeed the case is apparent from the recent work of Chung et al. [13]. There is abundant evidence that cyclin D is a key player in Restriction Point regulation (reviewed [2, 10, 12, 22, 23]). Following mitogen removal, the level of cyclin D starts to decline straight away, but remains high enough for long enough to sustain the kinase activity of cyclin-dependent kinases 4 and 6 (CDK4,6) to the point where irreversible commitment ensues [13], this being close to the start of S phase. Because of this lag, apparent mitogen independence is acquired several hours *before* the actual point in the cell cycle when commitment occurs. In other words, the true point of no return is much later than the onset of mitogen independence. Increasingly, this point seems likely to coincide with the abrupt, switch-like inactivation of the Anaphase Promoting Complex/Cyclosome-CDH1 complex (APC/C^CDH1^), the ubiquitin ligase responsible for the proteolysis of many proteins required for the initiation of DNA synthesis [10, 13, 18, 24].

## Insensitivity to CDK4/6 inhibition as an indicator of Restriction Point passage

Given that acquisition of mitogen independence is such a poor indicator of the timing of cell cycle commitment, a better measure might be the point of insensitivity to CDK4,6/cyclin D inhibitors such as palbociclib, ribociclib and abemaciclib [25]. These can block entry into DNA synthesis even when added late in G1 [13, 26–28] and on the face of it indicate the point when commitment becomes independent of CDK4,6/cyclin D (hereafter CDK4,6/D). This is some while after RB becomes fully phosphorylated and at a time when cyclin E (and CDK2/cyclin E activity) is already present [13, 26, 28]. However, the level of CDK2/cyclin E (hereafter CDK4,6/E) activity is evidently not yet sufficient to sustain RB hyperphosphorylation without the participation of CDK4,6/D [13, 28]. Thus, following palbociclib addition, RB rapidly becomes dephosphorylated (within 15 min) in cells that are not yet committed to cell cycle entry [13, 26, 28].

Although the point of insensitivity to palbociclib and similar inhibitors would seem like an objective, easily measurable indicator of when cell cycle commitment becomes independent of CDK4,6/D, it must nevertheless be noted that the action of such drugs is not straightforward. Recent studies [29] indicate that the complex of CDK4,6/D with the CDK inhibitor p27 (CDKN1B), when the p27 is phosphorylated on Tyr74, has RB kinase activity that is not inhibited by palbociclib. However, the kinase activity of the p27-containing trimer towards RB peptides containing an intact C-terminal tail is much less than the CDK4,6/D dimer and may be sufficient only to mono-phosphorylate RB [10, 30]. Instead, hyperphosphorylation of RB seems likely to be accomplished by the CDK4,6/D dimer, which is sensitive to palbociclib [29]. In contrast to p27, the complex between p21 (CDKN1A) and CDK4,6/D has no RB kinase activity since p21 is unable to undergo the activating tyrosine phosphorylation, lacking the equivalent of Tyr 74 found in p27 [29]. However, importantly, palbociclib causes the rapid dissociation of the p21 from the trimer and its relocation to and inhibition of CDK2/E [26]. (Note that p21, complexed with CDK2/E, remains in an inhibitory conformation even when, in response to mitogen-stimulation, the p21 is phosphorylated on a conserved tyrosine that sits in the active site of CDK2 [31]). Although not essential for cell cycle arrest by palbociclib and similar inhibitors [27, 28], the redistribution of p21 from CDK4 to CDK2 may contribute to the rapid fall in CDK2/E activity in cells not yet committed to cell cycle entry, seen following palbociclib treatment [10, 13, 26, 28]. Thus, the rapid reversal of RB hyperphosphorylation induced by palbociclib late in G1 may be due not only to direct inhibition of CDK4,6/D but also indirect inhibition of CDK2/E through displacement of p21 from CDK4,6/D. Put another way, RB hyperphosphorylation may be dependent both on the kinase activity of CDK4,6/D and on the ability of CDK4,6/D to sequester p21 away from CDK2/E so that it too can contribute to RB hyperphosphorylation. The point of insensitivity to palbociclib therefore marks the moment when both roles of CDK4,6/D are no longer needed, and when CDK2 activity alone becomes sufficient to sustain RB hyperphosphorylation – a common molecular definition of Restriction Point passage.

## So what is commitment?

Following mitogen-induced activation of CDK4,6/D, RB is phosphorylated on multiple sites leading in turn to a rise in E2F-dependent transcription, cyclin E expression (an E2F target) and a steady increase in CDK2/E activity [13, 18, 28]. This reinforces RB phosphorylation and further promotes E2F activation and the continuing rise in CDK2/E activity. Eventually, CDK2 activity reaches a critical threshold after which CDK4,6/D is no longer required [13, 28]. Thereafter, CDK2 activity alone is sufficient to drive RB phosphorylation and cyclin E expression in what is often assumed to be a manifestation of the positive-feedback loop (CDK2 → RB phosphorylation → E2F → cyclin E → CDK2) that confers theoretical bistability to the so-called RB-E2F switch [12]. Tripping this switch may represent the point of irreversible cell cycle commitment. However, the threshold of CDK2 activity needed to trigger independence from CDK4,6 is reached close in time to the abrupt inactivation of the E3 ubiquitin ligase APC/C^CDH1^ responsible (amongst other things) for the destruction of cyclin A and several factors required for DNA synthesis [13, 28]. Key to the switch-like inactivation of APC/C^CDH1^ is EMI1, another E2F target, together with CDK2 activity [18, 24, 32]. At low concentrations, EMI1 is a substrate of APC/C^CDH1^, which accordingly keeps its concentration low. However, phosphorylation by CDK2 inhibits APC/C^CDH1^. As CDK2 activity rises, it begins to suppress APC/C^CDH1^, allowing some EMI1 to escape degradation, accumulating to the point where it switches from being a substrate to an inhibitor, shutting off APC/C^CDH1^ irreversibly [24]. Following the abrupt inactivation of APC/C^CDH1^, cyclin A starts to accumulate, contributing to a further rise in CDK2 activity and RB phosphorylation [18, 32]. In addition, SKP2, a substrate-adaptor of the SCF ubiquitin ligase, is also stabilised, leading to the targeting of CDK-inhibitors p21, p27 and p57 for degradation [32, 33], potentially increasing the level of CDK2 activity yet further. It is conceivable that these additional inputs (cyclin A increase and p21/p27/p57 loss) are necessary for CDK2 activity to reach the level needed to supplant the requirement for CDK4,6/D activity (and mitogenic stimulation) in driving RB phosphorylation and E2F activation. If so, then the RB-E2F and APC/C^CDH1^ bistable switches are not separate transitions as usually described [10] but part of a single commitment step. Further work is required to clarify this important issue.

## Concluding remarks

As discussed here, palbociclib and similar inhibitors are effective in blocking entry into S phase even when added very late in G1, within an hour or so of S phase. As such, they provide a good indication of the point when cell cycle progression becomes independent of the mitogen-stimulated processes regulating CDK4,6/D activity, a point which may be regarded as equivalent to passage of the Restriction Point. Nevertheless, on removal of the inhibitor there is a lag of around 5 h before the arrested cells resume entry into S phase [25]. While this lag is much shorter than that shown by quiescent cells responding to mitogen restoration, it is nevertheless clear that cells do not remain poised close to the G1/S transition in the presence of palbociclib but instead slip back to some earlier point or state in G1. The precise nature of this point of arrest and what exactly has to be recapitulated before S phase entry, is clearly a matter of some importance.

## Data Availability

The data analysed here are freely available in the publication cited [14].

## Abbreviations

RB: Retinoblastoma tumour suppressor protein
CDK: Cyclin-dependent kinase
CDK4,6/D: Cyclin-dependent kinases 4 and 6 complexed with cyclin D
CDK2/E: Cyclin-dependent kinase 2 complexed with cyclin E.
APC/C^CDH1^: Anaphase promoting complex/cyclosome complexed with CDH1. a substrate-recognition subunit
